# Rare liver diseases—Etiology, diagnosis and management: A review

**DOI:** 10.17305/bb.2025.13278

**Published:** 2025-11-12

**Authors:** Qi Long, Rawaz D Tawfeeq, Yu Jiang, Huabao Liu, Meiao Tan

**Affiliations:** 1 Department of Respiratory and Critical Care Medicine, Chongqing Traditional Chinese Medicine Hospital, Chongqing, China; 2 Department of Clinical Analysis, College of Pharmacy, Hawler Medical University, Erbil, Iraq; 3 School of Public Health, Guangdong Pharmaceutical University, Guangdong, China; 4 Department of Hepatology, Chongqing Traditional Chinese Medicine Hospital, Chongqing, China

**Keywords:** Primary biliary cholangitis, primary sclerosing cholangitis, autoimmune hepatitis, gene therapy, precision medicine

## Abstract

Rare liver diseases (RLDs) are diverse and often misdiagnosed conditions that impose significant clinical and public health challenges due to their variable presentations and limited treatment options. This study aims to synthesize contemporary evidence on the etiology, classification, diagnostics, and management of RLDs and to identify near-term research and implementation priorities. We conducted a systematic search of PubMed and Scopus for the years 2015–2025 using predefined keywords. We included peer-reviewed human studies, such as guidelines, randomized trials, and large registries, focusing on mechanisms, diagnostic strategies, and treatments. We excluded animal studies and non-peer-reviewed reports, extracting data on disease biology, diagnostic tools, outcomes, and molecular therapies. RLDs can be categorized into genetic/inherited, autoimmune cholestatic, and other vascular/metabolic entities. Care for these diseases is increasingly guided by structured pathways that integrate biochemistry and serology with magnetic resonance cholangiopancreatography (MRCP), elastography, targeted next-generation sequencing (NGS), and selective biopsy. Emerging biomarkers, such as circulating microRNAs, alongside machine learning in imaging techniques, enhance disease staging and prognostication. Key management strategies include the use of bile-acid modulators, surgical interventions, and ileal bile acid transporter (IBAT) inhibitors for progressive familial intrahepatic cholestasis (PFIC). Lifelong copper chelation is recommended for Wilson disease, with trientine preferred for neurologic phenotypes. Supportive care in alpha-1 antitrypsin deficiency (A1ATD) is complemented by the investigation of molecular chaperones. Additionally, gene-directed therapies, gene editing, RNA-based approaches, and cell therapies show early promise but raise concerns regarding durability, safety, and ethical considerations, particularly for pediatric patients. In conclusion, implementing precision medicine frameworks that rely on standardized diagnostics, multicenter registries, and equitable access is crucial for facilitating earlier detection and translating mechanism-targeted therapies into sustainable, globally accessible benefits.

## Introduction

Rare liver diseases (RLDs) represent a subset of hepatic disorders that pose significant clinical and public health challenges due to diagnostic inaccuracies, variable presentations, and limited treatment options. A disease is classified as rare if it affects fewer than 200,000 individuals in the United States or fewer than 1 in 2000 people in Europe [1]. Although RLDs are uncommon, frequent misdiagnosis can lead to liver injury before detection [2, 3]. Conditions such as Wilson disease (WD), alpha-1 antitrypsin deficiency (A1ATD), progressive familial intrahepatic cholestasis (PFIC), and primary biliary cholangitis (PBC) stem from distinct genetic, metabolic, or autoimmune defects [4–7]. Some of these diseases manifest early in life and progress rapidly without intervention, while others remain asymptomatic until advanced stages. WD is an autosomal recessive disorder affecting copper metabolism, resulting in hepatic, neurological, and psychiatric symptoms [4, 8, 9]. A1ATD predominantly affects children and young adults and is frequently undiagnosed [10–12]. PFIC encompasses inherited cholestatic conditions arising from mutations in hepatocanalicular transporter genes, leading to persistent cholestasis and cirrhosis [13–15]. Autoimmune cholestatic diseases, such as PBC, cause progressive damage to intrahepatic bile ducts and are increasingly reported in global populations beyond Western regions [7, 16, 17]. Advances in molecular diagnostics, next-generation sequencing (NGS), and disease registries have enhanced disease classification. Management strategies include pharmacologic therapy, transplantation, and supportive care, with outcomes influenced by underlying pathology [1, 2, 18]. Innovations in gene-based therapies, targeted biologics, and updated diagnostic strategies facilitate earlier detection and personalized treatment [19, 20]. This review aims to discuss clinically relevant RLDs, their underlying mechanisms, recent therapeutic advancements, and future research directions to enhance patient outcomes.

## Methods and study design

An exhaustive search strategy was employed to gather published literature on RLDs. Articles published between 2015 and 2025 were systematically searched in PubMed and Scopus using various keywords, including “*rare liver diseases*,” “*Wilson disease*,” “*alpha-1 antitrypsin deficiency*,” “*progressive familial intrahepatic cholestasis*,” “*primary biliary cholangitis*,” “*primary sclerosing cholangitis*,” “*autoimmune hepatitis*,” “*gene therapy*,” “*RNA therapy*,” and “*precision medicine*.” Articles were included if they addressed the etiology, diagnostic strategies, or management approaches for specific RLDs or provided clinical data pertinent to therapeutic or molecular advancements. Animal studies, non-peer-reviewed reports, and irrelevant publications were excluded. Data extraction focused on disease mechanisms, diagnostic tools, treatment outcomes, and molecular therapies, prioritizing evidence from clinical guidelines, randomized trials, and large registry-based studies.

## Classification of RLDs

RLDs can be categorized into three primary groups: genetic or inherited disorders, autoimmune liver diseases, and a variety of structurally or metabolically distinct conditions (Table 1).

**Table 1:** Classification of rare liver diseases with significant features

**Classification RLDs**	**Disease name**	**Pathophysiology**	**Clinical presentation**	**Diagnosis**	**Management**	**References**
Genetic/inherited	Wilson’s disease	Defective *ATP7B* gene causing copper accumulation	Hepatic failure, neuropsychiatric manifestations	Serum ceruloplasmin, 24-h urinary copper, liver biopsy	Chelating agents (D-penicillamine), zinc therapy, liver transplantation	[4, 9, 29]
	Alpha-1 antitrypsin deficiency	Mutated *SERPINA1* gene leading to hepatic AAT retention	Neonatal hepatitis, cirrhosis, hepatocellular carcinoma	Serum AAT levels, genotyping, liver biopsy	Supportive care; liver transplantation if decompensated	[30, 31]
	Progressive familial intrahepatic cholestasis (pfic)	Mutations in bile transporter genes impairing bile flow	Infantile cholestasis, pruritus, progressive liver failure	Liver function tests, genetic analysis	Medical therapy, partial biliary diversion, liver transplantation	[32, 33]
	Hereditary hemochromatosis	*HFE* gene mutation leading to iron overload	Fatigue, cirrhosis, diabetes mellitus, cardiomyopathy	Serum ferritin, transferrin saturation, *HFE* genotyping	Phlebotomy, iron chelation therapy	[21, 34]
	Alagille syndrome	*JAG1* or *NOTCH2* mutations causing bile duct paucity	Cholestasis, cardiac anomalies, butterfly vertebrae	Clinical assessment, genetic testing, liver biopsy	Nutritional optimization, liver transplantation in advanced disease	[22]
Autoimmune	Autoimmune hepatitis (AIH)	T-cell-mediated destruction of hepatocytes	Fatigue, jaundice, transaminase elevation	Autoantibody profile, serum IgG, liver biopsy	Corticosteroids, azathioprine	[35, 36]
	Primary biliary cholangitis (PBC)	Autoimmune destruction of intrahepatic bile ducts	Pruritus, fatigue, cholestatic pattern	Antimitochondrial antibody (AMA), elevated ALP	Ursodeoxycholic acid, obeticholic acid, seladelpar	[7, 24]
	Primary sclerosing cholangitis (PSC)	Chronic inflammation and fibrosis of intra- and extrahepatic bile ducts	Cholestasis, association with IBD, risk of cholangiocarcinoma	MRCP, liver biopsy (if indicated)	No curative drug therapy; liver transplantation in advanced disease	[27, 37]
Other rare conditions	Budd–Chiari syndrome	Hepatic vein thrombosis causing venous outflow obstruction	Ascites, hepatomegaly, liver failure	Doppler ultrasonography, CT/MRI venography	Anticoagulation, TIPS, surgical shunts	[38]
	Congenital hepatic fibrosis	Ductal plate malformation with periportal fibrosis	Portal hypertension, splenomegaly	Imaging, liver histology	Supportive care; management of portal hypertension	[39]
	Glycogen storage disease (hepatic types)	Enzyme defects in glycogen metabolism	Hypoglycemia, hepatomegaly, growth retardation	Enzyme assay, genetic testing, metabolic screening	Dietary modification, surveillance for hepatic adenomas	[40–42]
	LAL-D	*LIPA* gene mutation causing lysosomal lipid accumulation	Hepatosplenomegaly, dyslipidemia, liver fibrosis	LAL enzyme assay, *LIPA* genotyping	Enzyme replacement therapy (sebelipase alfa)	[43]

Abbreviations: WD: Wilson disease; A1ATD: Alpha-1 antitrypsin deficiency; PFIC: Progressive familial intrahepatic cholestasis; AIH: Autoimmune hepatitis; PBC: Primary biliary cholangitis; PSC: Primary sclerosing cholangitis; AAT: Alpha-1 antitrypsin; AMA: Anti-mitochondrial antibody; ALP: Alkaline phosphatase; IgG: Immunoglobulin G; MRCP: Magnetic resonance cholangiopancreatography; CT: Computed tomography; MRI: Magnetic resonance imaging; IBD: Inflammatory bowel disease; TIPS: Transjugular intrahepatic portosystemic shunt; ATP7B: ATPase copper-transporting beta; SERPINA1: Serpin family A member 1; HFE: Homeostatic iron regulator; JAG1: Jagged 1; NOTCH2: Notch receptor 2; LAL-D: Lysosomal acid lipase deficiency; LAL: Lysosomal acid lipase; LIPA: Lipase A, lysosomal acid type.

**Figure 1.**
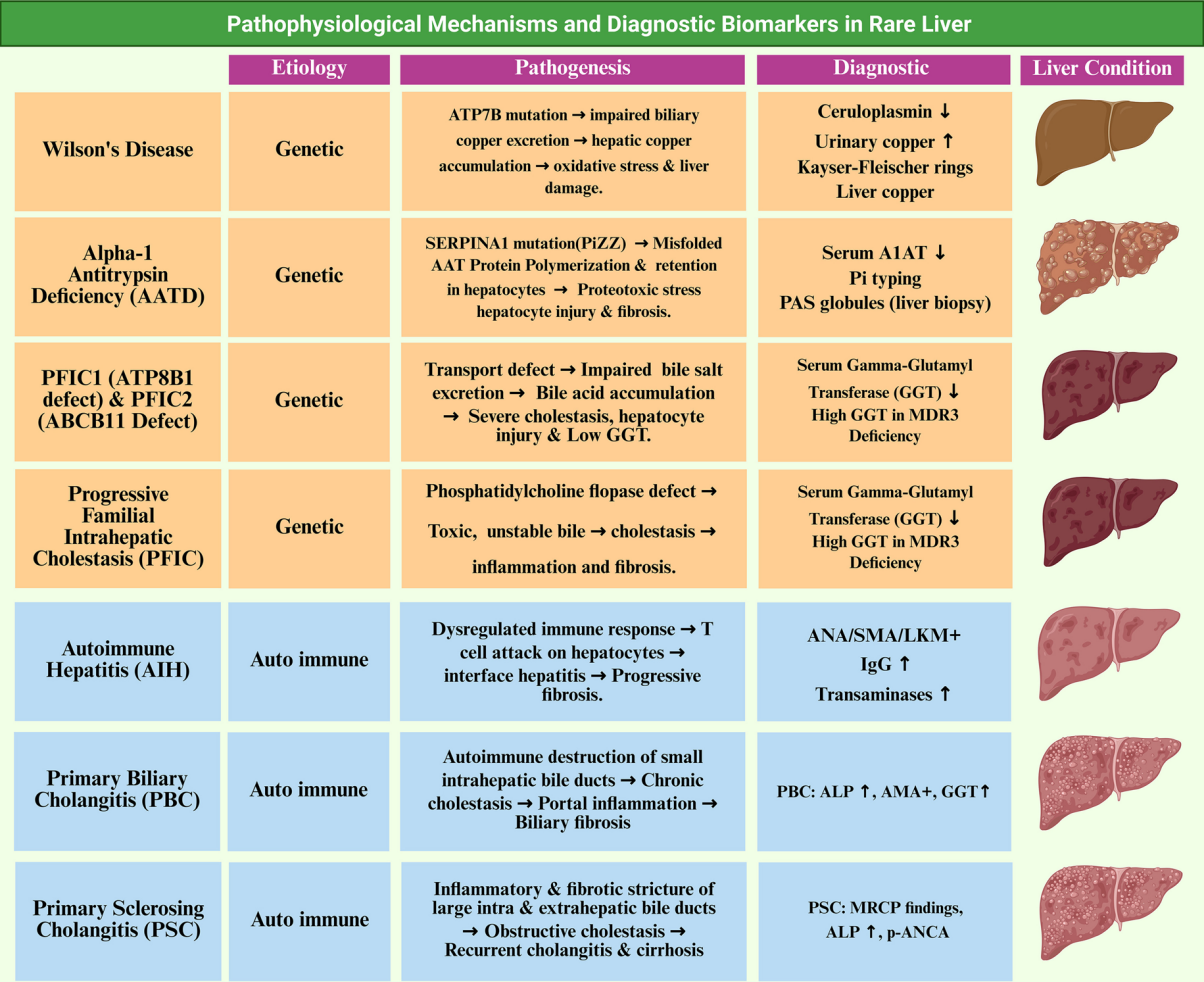
**Pathophysiological mechanisms and diagnostic biomarkers in rare liver diseases.** This schematic provides an overview of the key pathogenic pathways and diagnostic biomarkers that facilitate early detection and monitoring of rare liver diseases. It highlights the role of modifier genes and epigenetic regulators in influencing disease severity, phenotypic variability, and therapeutic responses.

### Genetic and inherited liver disorders

WD is caused by mutations in the *ATP7B* gene, disrupting copper transport and resulting in toxic accumulation in the liver, brain, and other organs. Patients may experience hepatic failure, psychiatric changes, or neurological deterioration. Initial diagnosis, through serum ceruloplasmin measurement, 24-h urinary copper estimation, and liver biopsy, is crucial for initiating chelation or zinc therapy, as delayed recognition is common due to variable clinical presentations in adolescents and young adults [4, 8, 9]. A1ATD, an inherited liver disorder, arises from *SERPINA1* mutations that inhibit the secretion of alpha-1 antitrypsin (AAT) protein, leading to its retention in hepatocytes and chronic liver injury. While pulmonary involvement is frequent, hepatic disease is often overlooked. Patel and Teckman [11] reported low awareness of A1ATD as a cause of neonatal or chronic hepatitis and cirrhosis in PiZZ homozygotes. Management is primarily supportive, with liver transplantation being the only curative option in advanced cases [6, 12]. PFIC includes autosomal recessive disorders resulting from mutations in *ATP8B1*, *ABCB11*, or *ABCB4* genes, leading to impaired bile secretion, early-onset cholestasis, pruritus, and progression to cirrhosis [14, 15]. Genetic testing has improved diagnosis and enhanced genotype–phenotype correlation. Intractable cases may necessitate surgical biliary diversion or liver transplantation. Hemochromatosis encompasses genetic disorders characterized by excessive intestinal iron absorption and hepatic iron deposition, resulting in fibrosis, cirrhosis, and hepatocellular carcinoma (HCC) [21]. Most cases involve *HFE* mutations, although non-*HFE* variants also contribute. Alagille syndrome, primarily caused by JAG1 mutations and rarely by NOTCH2 mutations, presents with reduced intrahepatic bile ducts, cholestasis, cardiac defects, facial dysmorphism, and vertebral anomalies [22, 23]. Symptoms typically appear in infancy, with diagnosis confirmed through genetic testing. Management focuses on symptomatic control and liver transplantation for progressive disease.

### Autoimmune liver diseases

Autoimmune liver diseases comprise a group of rare hepatic conditions primarily affecting adults. Autoimmune hepatitis (AIH) is characterized by immune-mediated hepatocyte destruction, accompanied by elevated aminotransferases, hypergammaglobulinemia, and interface hepatitis on biopsy. Diagnosis necessitates the exclusion of viral and metabolic etiologies, along with the presence of positive autoantibody reports. Many patients respond favorably to corticosteroids and azathioprine; however, some exhibit resistance and require alternative immunosuppressive agents [2].

PBC involves the progressive destruction of intrahepatic bile ducts, resulting in cholestasis and cirrhosis. Tanaka [7] noted its increasing prevalence in both Eastern and Western populations, particularly among middle-aged women. The primary diagnostic marker is antimitochondrial antibodies (AMAs). Ursodeoxycholic acid (UDCA) is the standard treatment, though 30%–40% of patients experience incomplete response and necessitate obeticholic acid (OCA) [16, 24]. Recent guidelines have emphasized the importance of early stratification and integrated care pathways to enhance treatment outcomes [24, 25]. In advanced cases, these guidelines also address the management of complications, such as portal hypertension, with portal vein thrombosis (PVT) being a significant concern in cirrhosis [26]. Primary sclerosing cholangitis (PSC) is a chronic fibroinflammatory disorder affecting both intrahepatic and extrahepatic bile ducts, often associated with inflammatory bowel disease. The disease progresses unpredictably, and there are currently no effective drug therapies available. The absence of reliable biomarkers and disease-modifying treatments renders PSC one of the most challenging conditions in autoimmune hepatology [27].

### Other rare liver conditions

Few liver diseases present as significant clinical challenges due to their severity and diagnostic complexity as Budd-Chiari syndrome. This condition arises from hepatic venous outflow obstruction, typically due to thrombosis. Clinical presentation ranges from asymptomatic hepatomegaly to fulminant hepatic failure. Diagnosis relies on Doppler ultrasonography or CT venography, with treatment determined by disease severity [1]. PVT is a vascular complication frequently observed in cirrhosis and other prothrombotic states. It causes thrombotic occlusion of the portal vein, leading to portal hypertension, variceal bleeding, and ascites. Diagnosis is achieved through Doppler ultrasound or cross-sectional imaging, and treatment focuses on anticoagulation and addressing underlying causes [26]. Congenital hepatic fibrosis is a developmental liver disorder characterized by periportal fibrosis and abnormal ductal plate formation. It often coexists with polycystic kidney disease and typically presents in childhood with portal hypertension, splenomegaly, or variceal bleeding. Liver function tends to remain stable until complications arise [28]. Glycogen storage diseases (types I and III) result from enzyme deficiencies that cause hepatomegaly, hypoglycemia, and growth delays. Nutritional therapy remains the standard treatment, with gene therapy currently under investigation [1, 28].

## Pathophysiological mechanisms and disease origin

The pathogenesis of RLDs is influenced by genetic mutations that disrupt metabolic and transport pathways, as well as dysregulated immune responses against hepatic tissue. These genetic mutations result in altered protein function and disrupted cellular homeostasis. Impaired enzyme activity, defective transport proteins, and the accumulation of toxic compounds drive disease progression. Figure 1 illustrates the primary pathogenic mechanisms and diagnostic biomarkers for early detection and monitoring. Modifier genes and epigenetic factors, including DNA methylation and microRNAs, play roles in determining disease severity, clinical variability, and treatment response [1, 19].

### Copper metabolism diseases

WD, caused by mutations in the *ATP7B* gene, leads to copper accumulation, resulting in hepatocyte and mitochondrial damage, hepatomegaly, fibrosis, and cirrhosis. Once the liver can no longer store copper, it enters the bloodstream and accumulates in other organs, particularly the basal ganglia, leading to neuropsychiatric symptoms. Impaired copper transport and inadequate antioxidant defenses regulate the disease process, with modifier genes influencing its severity and clinical presentation [4, 9, 44].

### Protein folding and aggregation disorders

A1ATD results in the accumulation of misfolded AAT polymers within hepatocytes. These deposits induce endoplasmic reticulum stress and activate the unfolded protein response, leading to oxidative stress, inflammation, and fibrosis [45]. This process increases the risk of HCC. Clinical manifestations range from neonatal hepatitis to cirrhosis in adults [31, 46]. Genetic factors, including defects in the endoplasmic reticulum-associated degradation (ERAD) pathway and the HFE-H63D variant, contribute to disease severity and clinical expression [47].

### Bile transport and cholestatic disorders

PFIC encompasses three genetic subtypes (types 1–3) caused by mutations in bile salt transporter genes *ATP8B1*, *ABCB11*, and *ABCB4*. Impaired bile acid transport results in accumulation within hepatocytes, leading to cellular injury, chronic cholestasis, and severe pruritus [13, 33]. Early surgical biliary diversion or the use of ileal bile acid transporter inhibitors can delay disease progression; however, many affected children ultimately progress to end-stage liver disease, necessitating transplantation [15, 23, 48].

### Iron overload syndromes

Hereditary hemochromatosis is characterized by mutations in the *HFE* gene, which disrupt the regulation of hepcidin and ferroportin, leading to excessive intestinal iron absorption. This results in the accumulation of iron deposits in hepatocytes, causing oxidative injury, activation of hepatic stellate cells, and subsequent progression to fibrosis and cirrhosis [22]. Variants in other genes, including *HJV*, *HAMP*, and *TFR2*, also contribute to the disease, resulting in varying degrees of iron loading and organ damage [22, 34].

### Autoimmune liver diseases

Autoimmune liver diseases encompass AIH, PBC, and PSC. AIH is characterized by T cell-mediated injury at the portal-parenchymal interface, leading to hypergammaglobulinemia and the presence of autoantibodies, such as ANA, SMA, and LKM1. Common clinical manifestations include fatigue, jaundice, and elevated aminotransferase levels [36]. PBC is caused by immune-mediated destruction of intrahepatic bile ducts, specifically targeting the pyruvate dehydrogenase complex, and is strongly associated with anti-mitochondrial antibodies [7, 24]. PSC is marked by chronic inflammation and fibrosis of the bile ducts, often co-occurring with inflammatory bowel disease [27]. The pathogenesis of these diseases is influenced by a loss of immune tolerance, shaped by genetic predisposition, environmental factors, and epigenetic modifications, as well as signals from the gut-liver axis [35, 49].

## Clinical presentation and disease-specific features

RLDs exhibit considerable genetic and pathological variability, often manifesting symptoms that delay diagnosis. Common symptoms include fatigue, pruritus, jaundice, and hepatomegaly. Severe, treatment-resistant pruritus is particularly prevalent in PFIC and PBC. Intrahepatic bile retention may lead to right upper quadrant pain and liver injury. Neurological, hematological, and dermatological manifestations, such as tremors, cognitive changes, and xanthomas, can accompany hepatic conditions. In WD, tremors and behavioral changes frequently precede hepatic symptoms [4, 9]. PFIC typically presents in infancy with pruritus and growth failure [15, 33], while A1ATD may cause neonatal jaundice or remain undiagnosed until adulthood [11, 46].

### Biochemical and immunological diagnostics

Diagnosing RLDs necessitates a comprehensive approach that integrates clinical, biochemical, serological, and imaging findings. In WD, a low ceruloplasmin level, elevated urinary copper, and hepatic copper measurement confirm the diagnosis, while MRI often reveals basal ganglia hyperintensities associated with neurological symptoms [4, 9]. Disease-specific markers facilitate diagnosis: low serum AAT levels with phenotyping in A1ATD [5, 6]; increased aminotransferases, IgG, and autoantibodies in AIH [36]; elevated ALP with anti-mitochondrial antibodies in PBC [7, 24]; and characteristic cholangiographic findings in PSC [27]. In A1ATD, periodic acid–Schiff (PAS)-positive, diastase-resistant inclusions identified through gene panel or whole-exome sequencing enable early and accurate diagnosis, particularly in pediatric or atypical cases, thereby reducing the need for liver biopsy [5, 11]. Biopsy remains valuable for histological confirmation, disease staging, and assessing treatment response when non-invasive tests are inconclusive [15, 50]. AIH typically presents with elevated transaminases, increased IgG levels, and specific autoantibodies such as ANA, SMA, or LKM; the revised IAIHG criteria assist in diagnosis and grading [36]. PBC is characterized by elevated ALP and M2-type AMA, often accompanied by increased GGT [7, 24]. PSC is defined by cholangiographic evidence of multifocal bile duct strictures and a beaded appearance of the ducts on MRCP or ERC. Imaging techniques, including ultrasound, elastography, MRI, and MRCP, support diagnosis and ongoing monitoring [26, 27, 50].

**Figure 2.**
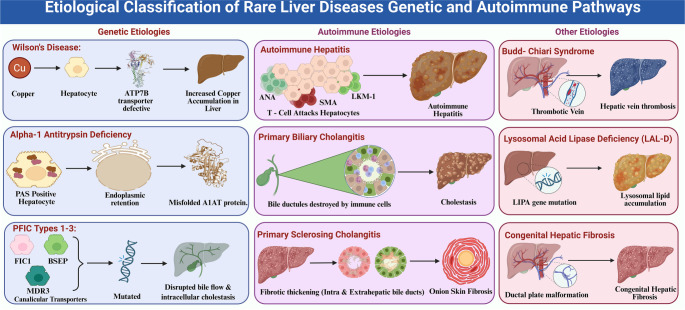
**Etiological classification of rare liver diseases: Genetic and autoimmune pathways.** The panels illustrate representative disorders within the genetic (Wilson disease, A1ATD, PFIC1–3) and autoimmune (AIH, PBC, PSC) categories, highlighting key molecular defects and characteristic lesions. Abbreviations: A1ATD: Alpha-1 antitrypsin deficiency; PFIC: Progressive familial intrahepatic cholestasis; AIH: Autoimmune hepatitis; PBC: Primary biliary cholangitis; PSC: Primary sclerosing cholangitis.

## Etiology and management of RLDs

The etiologies of RLDs can be broadly classified into genetic, autoimmune, and other uncommon causes, each characterized by specific mechanisms, clinical courses, and therapeutic considerations that inform patient management strategies (Figure 2).

### Genetic etiologies

Several RLDs result from single-gene mutations, with WD serving as a prominent example. Mutations in the *ATP7B* gene disrupt biliary copper excretion, leading to copper accumulation in the liver and progressive injury [4, 8]. Diagnosis can be challenging due to the variability in clinical presentations. Chanpong and Dhawan [51] reported that approximately 30% of children present with acute liver failure, often without Kayser–Fleischer rings, highlighting the importance of genetic testing. Standard treatment involves prolonged chelation therapy with D-penicillamine or trientine, although the choice of initial drug is debated, as D-penicillamine can exacerbate neurological symptoms in some patients [52]. NGS has enhanced diagnosis by directly identifying *ATP7B* mutations and recognizing atypical cases [44]. Recent studies are investigating small molecules, such as 4-phenylbutyrate, to correct misfolded ATP7B protein, and CRISPR/Cas9-based gene editing has demonstrated early success in experimental models [53, 54].

A1ATD is a protein misfolding disorder resulting from mutations in the *SERPINA1* gene, which lead to the accumulation of polymers in hepatocytes. This accumulation induces endoplasmic reticulum stress, inflammation, and progressive liver injury [45]. The hepatic impact is most pronounced in PiZZ homozygotes. Hamesch et al. [55] reported that 35% of adult Pi*ZZ individuals exhibited significant fibrosis (≥F2), and 9% had advanced fibrosis (≥F3), confirming the toxic effects of retained Z-variant protein. Clinically, A1ATD often manifests as unexplained neonatal cholestasis. Teckman et al. [46] noted a male predominance, with 73% of affected boys developing severe liver disease by adolescence. Liver transplantation remains the only curative treatment for end-stage disease [56], while supportive management is also essential. Joly et al. [47] identified HFE-H63D as a genetic modifier that exacerbates hepatic injury, elucidating the variability of the disease. Segeritz et al. [45] employed iPSC-derived hepatocyte models to investigate the unfolded protein response and inflammation, providing a platform for therapeutic testing. Ongoing studies are exploring RNA therapies aimed at reducing mutant protein production.

PFIC represents a group of autosomal recessive liver disorders caused by mutations in hepatocanalicular transport genes. PFIC1 (*ATP8B1*), PFIC2 (*ABCB11*), and PFIC3 (*ABCB4*) impair phospholipid translocation, bile salt export, and phosphatidylcholine secretion, leading to bile acid toxicity and liver injury [13, 14, 33]. Symptoms typically begin early, often in infancy, presenting as severe itching and poor growth. Genetic classification is crucial for prognosis and treatment planning. Vitale et al. [15] reported that surgical biliary diversion normalized bile acids and alleviated pruritus in over 80% of selected PFIC1 and PFIC2 patients, thereby preventing disease progression and reducing the need for transplantation. Davit-Spraul et al. [57] found that targeted genetic testing identified a molecular diagnosis in 68% of children with low-GGT PFIC. Established genotype–phenotype correlations indicate that *ABCB11* mutations resulting in loss of BSEP protein lead to rapid fibrosis and an increased risk of HCC [33, 48]. While liver transplantation is effective for end-stage disease, it is less successful in PFIC1 due to extrahepatic symptoms and recurrent steatohepatitis associated with the *ATP8B1* defect [15, 23]. Current research is focused on identifying modifier genes and epigenetic factors that may elucidate clinical variability and guide future individualized therapies.

### Autoimmune etiologies

AIH is a chronic immune-mediated liver disorder characterized by elevated aminotransferases, hypergammaglobulinemia, and the presence of autoantibodies such as ANA, SMA, or LKM-1. It is associated with the HLA-DR3 and HLA-DR4 alleles. Diagnosis necessitates the exclusion of viral or metabolic causes and confirmation of interface hepatitis through histological examination. First-line therapies include corticosteroids and azathioprine, while second-line immunosuppressants are reserved for refractory cases [36]. Additional factors, such as polymorphisms in CTLA-4 and TNF-α, may influence disease progression [58]. Biologic agents, including rituximab and immune checkpoint-targeted therapies, have broadened treatment options for resistant cases. Epigenetic mechanisms, including altered DNA methylation and microRNA expression, are being investigated as potential biomarkers of disease activity and therapeutic response [35].

PBC is a chronic autoimmune cholestatic liver disease characterized by the progressive destruction of small intrahepatic bile ducts. It predominantly affects middle-aged women and is strongly associated with AMAs. Bile duct injury results in bile stasis, hepatocellular damage, fibrosis, and eventual cirrhosis. UDCA is the established first-line therapy; however, 30%–40% of patients exhibit an incomplete biochemical response. OCA and fibrates are available as second-line treatments in these cases [7, 16, 24]. Epidemiological studies indicate an increasing incidence in Asian populations, highlighting the condition’s global distribution [17].

PSC is characterized by chronic inflammation and fibrosis of intra- and extrahepatic bile ducts, leading to multifocal strictures, cholestasis, and frequent associations with ulcerative colitis. MRCP is effective for identifying characteristic ductal irregularities [27]. Currently, no curative therapy exists, and liver transplantation is the only definitive option for advanced disease [49, 56]. The elevated risk of cholangiocarcinoma and other hepatobiliary cancers necessitates stringent surveillance and coordinated care. Genomic studies have linked HLA variants and loci such as FUT2 and MST1 to bile acid metabolism and immune regulation [27]. Ongoing research into molecular imaging, biomarkers, antifibrotic agents, and microbiome-based therapies aims to enhance early detection and treatment of RLDs. Furthermore, the integration of genetic and multi-omics data through registries aims to improve precision care and clinical outcomes [59–61].

## Diagnostic strategies and challenges in RLDs

### Clinical evaluation and initial laboratory testing

The diagnosis of RLDs begins with a clinical examination that assesses symptoms such as fatigue, jaundice, pruritus, hepatomegaly, or unexplained enzyme alterations. Initial laboratory evaluations, including liver function tests to assess hepatocellular injury, coagulation studies for hepatic synthesis, and cholestatic markers such as ALP and GGT, are essential [2, 19]. The presence of antinuclear (ANA), smooth muscle (SMA), antimitochondrial (AMA), and liver–kidney microsomal (LKM-1) antibodies can confirm autoimmune causes such as AIH or PBC [7, 36]. Overlapping biochemical patterns, including elevated transaminases and cholestatic markers, may complicate differentiation [1, 19]. The absence of specific autoantibodies necessitates a biopsy. Correlating these factors is critical for accurate diagnosis in RLDs.

### Genetic testing

Genetic testing is essential for diagnosing monogenic RLDs. NGS allows for the rapid identification of pathogenic variants, improving diagnostic accuracy and facilitating early therapeutic intervention. *ATP7B* sequencing confirms WD and facilitates family screening [4, 8, 9]. PFIC diagnosis relies on identifying mutations in *ATP8B1*, *ABCB11*, and *ABCB4*, guiding clinical decision-making from pharmacologic therapy to biliary diversion or transplantation [23, 33, 48]. NGS achieves a diagnostic success rate of up to 68% in low-GGT PFIC and other cholestatic disorders [57]. However, challenges remain in diagnosing RLDs due to structural variants, deep intronic changes, or complex reorganizations that may require additional approaches [1, 45]. Variant interpretation necessitates the integration of clinical phenotype, segregation analysis, and functional validation to avoid misclassification. Limited bioinformatics resources and restricted access to genetic counseling contribute to delays in diagnosis, particularly in low-resource settings [45].

### Imaging modalities

Imaging is essential for evaluating liver morphology, bile duct anatomy, and fibrosis staging in RLDs. Ultrasound is effective in detecting hepatomegaly, splenomegaly, and biliary dilation, but it lacks the resolution to identify small ducts. In contrast, magnetic resonance cholangiopancreatography (MRCP) provides radiation-free, high-quality visualization of cholangiopathies, such as PSC, revealing multifocal strictures and characteristic ductal beading [27]. Transient elastography (FibroScan) allows for bedside measurement of liver stiffness and assessment of fibrosis severity; however, its accuracy may decline in the presence of obesity, inflammation, or ascites [19, 62]. Gan et al. [19] noted that these limitations could lead to an underestimation of fibrosis stage, particularly in cholestatic liver disease where bile duct proliferation complicates stiffness measurements. Magnetic resonance elastography (MRE) facilitates comprehensive assessment of liver fibrosis with improved sensitivity and specificity, making it particularly valuable for PSC and advanced fibrotic disease. Nevertheless, high costs, limited availability, and lack of standardization across platforms impede the widespread use of MRE, as demonstrated in multicenter studies. Additional challenges include overlap with common hepatic disorders, poor detection of small-duct cholangiopathies or early fibrosis, and restricted access at many medical centers [19, 62].

### Histopathology and liver biopsy

Liver biopsy remains the gold standard for confirming diagnoses, staging disease, and excluding coexisting liver disorders. In WD, liver biopsy quantifies hepatic copper levels exceeding 250 µg/g dry weight, thereby confirming the diagnosis while also assessing inflammation and fibrosis in cases where biochemical or genetic results are inconclusive [4, 9, 44]. In autoimmune liver diseases, including AIH, PBC, and PSC, histological examination evaluates interface hepatitis, bile duct injury, and fibrosis, thereby guiding therapy in antibody-negative cases or partial treatment responders [7, 27, 36]. Biopsy is also instrumental in diagnosing A1ATD through the identification of characteristic PAS-positive, diastase-resistant hepatocytic inclusions [11, 30]. Despite its utility, liver biopsy is an invasive procedure associated with risks of bleeding, pain, and sampling error, necessitating the expertise of skilled hepatopathologists for accurate interpretation.

### Integrated diagnostic workflow

**Figure 3.**
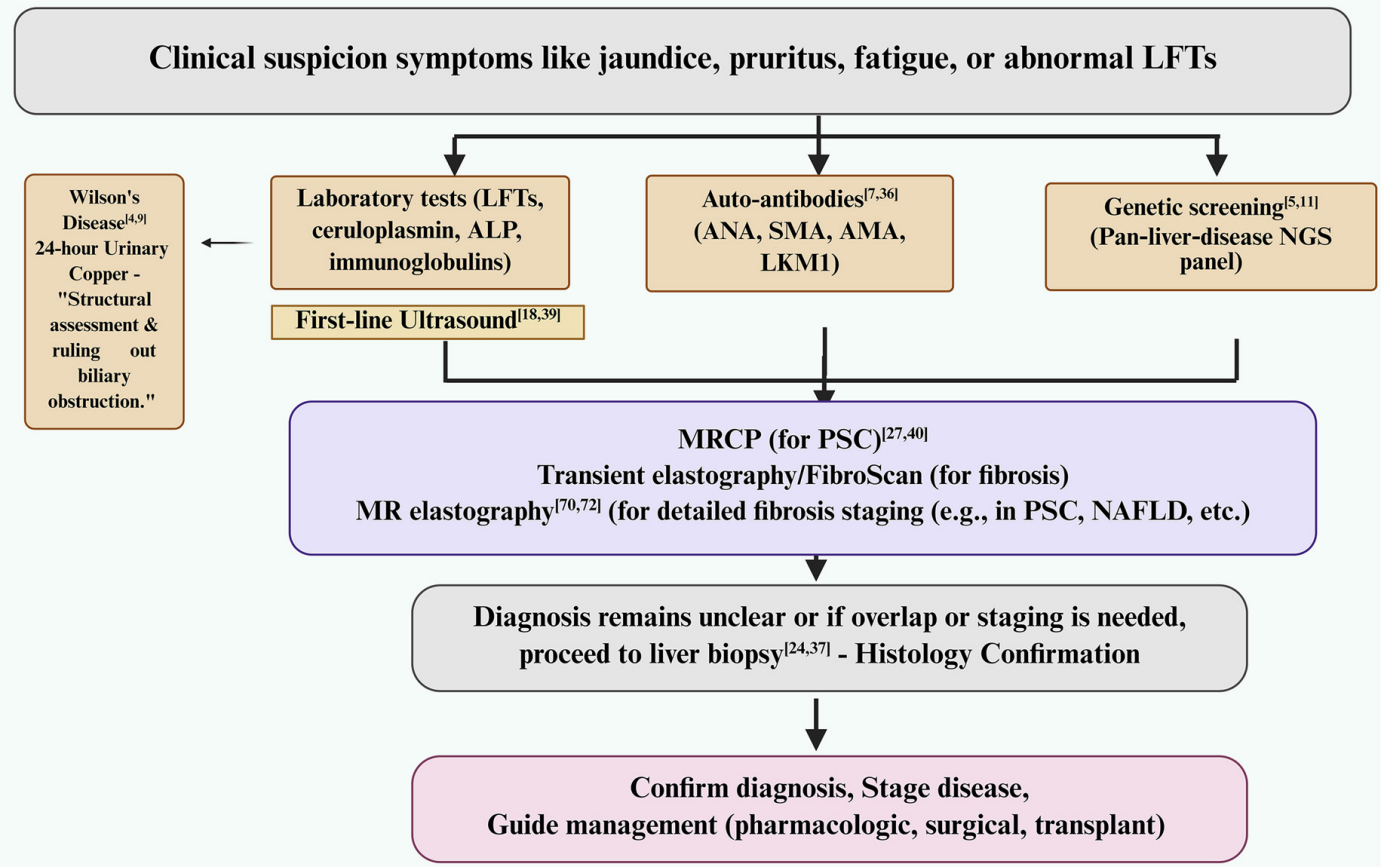
**Stepwise diagnostic algorithm for rare liver diseases.** From the initial clinical suspicion, the pathway incorporates baseline laboratory testing alongside first-line ultrasound, serologic autoantibody assessment, and targeted genetic screening. Cholangiopathy and fibrosis are evaluated using MRCP (for suspected PSC) and elastography—specifically transient elastography (FibroScan) and MR elastography. Testing for Wilson’s disease, through 24-h urinary copper measurement, is included when clinically indicated. If the diagnosis remains uncertain or if staging is necessary, a liver biopsy should be performed for histological confirmation. This process enables the confirmation of the diagnosis, staging of the disease, and guidance for pharmacological, surgical, or transplant management. Abbreviations: LFTs: Liver function tests; ALP: Alkaline phosphatase; ANA: Antinuclear antibody; SMA: Smooth muscle antibody; AMA: Anti-mitochondrial antibody; LKM1: Liver–kidney microsomal antibody type 1; NGS: Next-generation sequencing; MRCP: Magnetic resonance cholangiopancreatography; PSC: Primary sclerosing cholangitis; MR: Magnetic resonance; NAFLD: Non-alcoholic fatty liver disease.

A structured diagnostic algorithm enhances the accuracy of detecting RLDs and reduces unnecessary invasive procedures (Figure 3). This assessment includes a thorough patient history, clinical examination, and basic laboratory tests. Liver function tests evaluate hepatocellular injury, while elevated ALP or GGT levels indicate cholestasis. Autoantibody tests assist in identifying autoimmune diseases, and low ceruloplasmin levels are diagnostic for WD. Targeted NGS confirms monogenic disorders such as WD, PFIC, and A1ATD. MRCP delineates biliary anatomy, and elastography estimates liver fibrosis. A liver biopsy is warranted in cases of uncertain diagnoses, syndromes, or for evaluating inflammation and fibrosis. This comprehensive approach improves diagnostic precision, supports treatment planning, and minimizes the need for invasive procedures [2, 19].

### Emerging diagnostic advances and challenges

Novel diagnostic approaches are enhancing early identification of RLDs. Circulating microRNAs and cytokine techniques serve as non-invasive biomarkers for monitoring fibrosis and inflammatory activity, thereby reducing the necessity for repeated biopsies [51, 62]. Multi-omics methodologies, encompassing genomic, epigenomic, proteomic, and metabolomic data, facilitate precise molecular classification and target pathways relevant for appropriate treatment. Machine-learning-driven imaging tools improve the detection of structural liver changes, quantify fibrosis, and predict disease progression or therapeutic response with greater accuracy than traditional evaluation methods [61].

**Table 2:** Summary of current therapeutic and supportive management approaches in rare liver diseases

**Management approach**	**Description**	**Diseases**	**Key interventions**	**References**
Pharmacological treatments	Disease-specific therapies targeting underlying mechanisms or symptoms.	PBC, Wilson’s disease	UDCA improves cholestasis and delays progression (PBC); Obeticholic acid (FXR agonist) for UDCA non-responders (PBC); Copper chelators (D-penicillamine, trientine) and zinc for copper excretion (Wilson’s disease)	[7, 9, 26, 49, 52]
Nutritional management	Supplementation to correct malabsorption and prevent deficiencies associated with cholestasis.	PFIC, PBC	Fat-soluble vitamin (A, D, E, K) supplementation; MCTs to enhance caloric intake and absorption	[2, 15, 68]
Surgical interventions	Procedures to manage advanced liver damage and complications.	End-stage liver disease, Wilson’s disease, PFIC, A1ATD	Liver transplantation as definitive treatment; Biliary diversion to reduce pruritus and cholestasis; Post-transplant surveillance for recurrence or complications	[9, 15, 25, 49]
Multidisciplinary care	Collaborative management involving multiple specialties to address systemic and psychosocial aspects.	All RLDs	Coordination between hepatologists, geneticists, nutritionists, surgeons, and allied teams; Genetic counseling and family screening; Nutritional monitoring, especially in children; Psychological and educational support	[2, 19, 50, 72]
Pediatric considerations	Age-specific management strategies to address early disease onset and progression.	PFIC, Wilson’s disease, A1ATD	Early genetic testing and family screening; Nutritional optimization with vitamin and MCT supplementation; Early surgical evaluation or transplant when indicated	[15, 30, 46, 50]
Monitoring and surveillance	Continuous evaluation of disease activity and long-term complications.	All RLDs	Periodic liver biochemistry and elastography; HCC surveillance with ultrasound ± cross-sectional imaging in cirrhosis; Monitoring of therapy adherence, nutritional status, and psychosocial well-being	[12, 19, 26, 55, 56]

Abbreviations: RLDs: Rare liver diseases; PBC: Primary biliary cholangitis; PFIC: Progressive familial intrahepatic cholestasis; A1ATD: Alpha-1 antitrypsin deficiency; UDCA: Ursodeoxycholic acid; FXR: Farnesoid X receptor; MCT: Medium-chain triglycerides; HCC: Hepatocellular carcinoma.

## Current management strategies

In PBC, UDCA at a dosage of 13–15 mg/kg/day is the standard first-line therapy, effectively improving liver enzymes, slowing fibrosis progression, and extending transplant-free survival. The British Society of Gastroenterology/UK-PBC guidelines indicate that most patients adhering to UDCA achieve near-normal life expectancy [49]. Approximately 40% of patients exhibit an incomplete biochemical response (ALP >1.67 × ULN after 12 months) and may require additional therapy [26, 49]. OCA, a selective farnesoid X receptor (FXR) agonist, serves as a second-line treatment option. In the ENHANCE trial [37], 46% of patients receiving OCA 10 mg met the composite endpoint, compared to 10% in the placebo group. Its use is restricted in advanced cirrhosis due to the risk of hepatic failure and frequent pruritus requiring dose adjustments [37]. This has prompted the search for safer FXR agonists. A randomized study by Schramm et al. [63] indicated that tropifexor reduced ALP by a placebo-adjusted mean of -18.3% at a 60-µg dose, although pruritus remained common (45%). Peroxisome proliferator-activated receptor (PPAR) agonists offer an alternative mechanism. In the phase 3 ENHANCE trial, seladelpar achieved a --42.4% mean reduction in ALP at 3 months, with only a 14% incidence of pruritus, indicating better tolerability. However, high costs of emerging therapies continue to limit access for PBC patients in resource-constrained settings [19, 37].

In WD, lifelong copper chelation therapy is standard, though there is ongoing debate regarding the choice of the initial chelator. Mohr and Weiss [52] found that D-penicillamine is associated with a higher risk of early neurological worsening, leading to the preference for trientine in neurologically presenting cases. Gene therapy utilizing adeno-associated virus (AAV) vectors to deliver *ATP7B* (e.g., UX701) is under evaluation in early-phase clinical trials (Phase 1/2, NCT04884815). Pöhler et al. [53] demonstrated *in vitro* correction of *ATP7B* mutations using CRISPR/Cas9, which restored copper transport but exhibited limited editing efficiency in hepatocytes. RNA-based treatments remain experimental due to challenges related to delivery and durability. mRNA and gene-editing approaches face obstacles in achieving safe and reproducible hepatocyte delivery. Liver transplantation is the definitive treatment, yielding over 85% five-year survival in WD, underscoring the need for disease-modifying therapies to reduce reliance on transplantation [56, 64].

### Pediatric considerations

Early diagnosis and targeted treatment are critical in pediatric RLDs (Table 2). The identification of mutations in *ATP8B1*, *ABCB11*, and *ABCB4* informs therapeutic approaches in PFIC. Vitale et al. [15] reported that biliary diversion normalizes bile acids and alleviates pruritus in over 80% of PFIC1 and PFIC2 cases, thereby preventing the need for transplantation. WD may manifest in childhood as acute liver failure rather than the typical adolescent presentation. Chanpong and Dhawan [51] noted that approximately 30% of pediatric WD cases present in this manner, often with absent Kayser–Fleischer rings or low ceruloplasmin levels, necessitating *ATP7B* sequencing for confirmation. In A1ATD, neonatal cholestasis can mimic biliary atresia. Teckman et al. [46] showed that male children (73%) with early cholestasis are more likely to develop severe liver disease, supporting the need for early genetic testing and close monitoring to identify progression risk and guide timely interventions.

### Disease progression and monitoring

Many RLDs remain undetected until significant damage has occurred. In A1ATD, the hepatic accumulation of misfolded Z-variant protein induces cellular stress and fibrosis. Hamesch et al. [55] reported that in a large cohort of adult Pi*ZZ individuals, liver fibrosis was observed in 35% (*F* ≥ 2) and advanced fibrosis in 9% (*F* ≥ 3), confirming the direct hepatotoxicity of the retained protein. While ongoing surveillance is recommended, the most effective monitoring strategy remains uncertain. Tamber et al. [50] reviewed biomarker performance, noting that serum indices, including the ELF score, demonstrate varying reliability across different liver pathologies. Semi-annual ultrasound is standard practice but lacks sensitivity for HCC. Finotti et al. [56] advised that in A1ATD-related cirrhosis, combining ultrasound with cross-sectional imaging enhances early tumor detection and addresses the limitations of conventional screening methods.

## Emerging therapeutic approaches

### Gene therapy and gene editing

Gene therapy has evolved for the treatment of monogenic liver diseases, although safety and biological challenges restrict its clinical application. AAV vectors (AAV9) enable targeted delivery to hepatocytes, with UX701 aiming to restore copper regulation currently under clinical evaluation [37]. AAV genomes remain episomal, which can lead to transgene loss as hepatocytes divide, thereby limiting durability in pediatric populations [65, 66]. CRISPR-Cas9 technology offers a corrective approach by repairing mutations in genes such as *ATP7B* and *SERPINA1*, with successful *ATP7B* correction demonstrated by Pöhler et al. [53] in murine models [27, 32]. High-fidelity nucleases have improved editing accuracy; however, ongoing genomic monitoring and long-term safety evaluations are essential before routine clinical implementation [67].

### RNA-based therapeutics

Management of RLDs emphasizes targeted therapies. In WD, lifelong copper chelation, along with regular hepatic, neurological, and psychiatric monitoring, remains the standard approach [4, 9, 52]. A1ATD management focuses on preventing chronic liver injury, as no therapies currently correct protein misfolding; transplantation remains the only curative option for end-stage disease [12, 56]. PFIC and Alagille syndrome present overlapping challenges, necessitating multidisciplinary care for issues such as pruritus, malnutrition, and portal hypertension prior to surgical intervention or transplantation [25, 49, 68]. Recent studies have emphasized mechanism-based therapies. Gene therapy and genome editing techniques, including CRISPR/Cas9, have shown promise in repairing causal mutations in A1ATD and WD, with successful preclinical gene correction reported [34, 53, 66]. FXR and PPAR agonists (e.g., Seladelpar) have demonstrated improvements in cholestasis and inflammation in PBC [37, 63]. Continued progress relies on enhanced diagnostics, personalized care, and global registries that promote clinical precision [61, 69, 70].

### Cell-based therapies

Cell-based therapies are currently being investigated as alternatives or bridges to transplantation for RLDs. Hepatocyte transplantation can restore partial metabolic function in conditions such as Crigler–Najjar syndrome and urea cycle defects; however, challenges such as donor shortages, low engraftment rates, and limited duration of benefits persist [56]. Transplanted hepatocytes exhibit restricted proliferation and survival within fibrotic tissue, as noted in early studies [34, 69]. Induced pluripotent stem cell (iPSC) technology provides a renewable autologous source of hepatocyte-like cells. Segeritz et al. [45] demonstrated that iPSC-derived hepatocytes can model A1ATD, reduce misfolded protein accumulation, and enhance cell function, although concerns regarding maturation, tumor risk, and associated costs remain. Mesenchymal stem cells (MSCs) exhibit immunomodulatory, antifibrotic, and reparative effects. Verbeke et al. [71] reported benefits in experimental cirrhosis and acute-on-chronic liver failure; however, maintaining cell viability and long-term safety presents significant barriers to clinical application.

### Pharmacological innovations

New molecular therapies are being developed to target core mechanisms in RLDs, including protein misfolding, inflammation, and metabolic dysfunction (Table 2). In A1ATD, pharmacologic chaperones correct Z-variant SERPINA1 misfolding and reduce hepatic accumulation [11]. In cholestatic RLDs, FXR agonist OCA and PPAR agonist seladelpar have shown improvements in biochemical responses in PBC and PFIC [7, 37]. The APASL 2022 clinical guidance supports these pathway-specific treatments and encourages the development of immunomodulatory agents [17]. Additional candidates targeting oxidative stress, fibrogenesis, and immune checkpoints are also under development [19]. High-throughput screening and multi-omics approaches are accelerating the discovery of precise, mechanism-based therapies for RLDs.

### Precision medicine and pharmacogenomics

Precision medicine is transforming the management of RLDs by aligning treatments with each patient’s genetic and molecular profile. Detailed genotyping refines diagnosis, prognosis, and therapy selection. In WD, *ATP7B* genetic variants influence clinical presentation and response to chelation therapy; missense mutations often correlate with milder hepatic disease, while truncating variants are associated with earlier neurological onset and intolerance to D-penicillamine [4, 9, 52, 72]. Pharmacogenomic data contribute to dose optimization and prediction of adverse reactions. In autoimmune and cholestatic RLDs, gene polymorphisms affecting drug metabolism and immune pathways can modify therapeutic outcomes [63, 73]. In A1ATD, pharmacologic chaperones specifically target the misfolded Z-SERPINA1 protein [11]. The integration of multi-omics approaches—genomics, proteomics, metabolomics, and epigenomics—supports biomarker identification and provides mechanistic insights [7, 12, 51, 55]. Proteomic and metabolomic studies in A1ATD and PBC have revealed inflammatory and fibrotic pathways that are valuable for early detection and personalized disease management [61].

### Patient-centered outcomes and quality of life

RLDs affect multiple organ systems and lead to symptoms such as fatigue, pruritus, growth delay, and neurocognitive impairment, all of which diminish daily activities and overall quality of life [2, 19, 49]. These complications are particularly pronounced in children, resulting in significant emotional and social stress. Patient-reported outcomes (PROs) are utilized to evaluate the impact of the disease and treatment responses beyond biochemical markers [12, 19, 46]. The assessment of health-related quality of life (HRQoL) is essential for individualizing therapy and informing psychological, nutritional, and social interventions [19, 72]. Commonly used instruments include the PBC-40, which focuses on fatigue and itching in adults with PBC [7, 49]; the Itch Numeric Rating Scale (Itch-NRS) for assessing pruritus in PFIC, PBC, and PSC; and the SF-36, which evaluates overall health [30, 72]. The Chronic Liver Disease Questionnaire (CLDQ) assesses a range of hepatic symptoms [12, 19], while the Pediatric Quality of Life Inventory (PedsQL) measures HRQoL in pediatric patients with PFIC, WD, and A1ATD [1, 15, 50].

### Health economics and accessibility

Advancements in gene therapy and RNA-based treatments have enhanced options for managing RLDs; however, significant affordability and access challenges persist, particularly in low- and middle-income countries [1, 56]. Market-driven pricing models have resulted in unequal global distribution of these therapies, with many patients remaining reliant on established treatments such as chelation or UDCA, while access to advanced therapies is often confined to specialized centers [7, 21]. Addressing these disparities necessitates international funding support, equitable pricing systems, and the integration of RLD care within universal health programs [1, 74].

## Limitations and future directions

Early identification of RLDs remains a significant challenge due to their initial manifestations, which often overlap with common hepatic disorders, as well as the lack of standardized diagnostic protocols. Diseases such as A1ATD, PBC, and PFIC are frequently misdiagnosed as viral or metabolic liver diseases, resulting in delayed assessment and treatment [7, 12]. Current pharmacological approaches typically alleviate symptoms without halting disease progression, and access to molecular or gene-based therapies is limited by cost and availability [1, 19]. In the case of PBC, OCA reduces biochemical markers but is associated with dose-limiting pruritus, complicating long-term adherence to treatment [26, 37]. Recent studies emphasize the importance of personalized therapy informed by genetic and molecular data. Biomarkers such as circulating miRNAs, extracellular vesicles (EVs), and autoantibodies are being investigated for their potential in early diagnosis [51, 62]. Emerging multicenter registries and CRISPR-derived technologies are anticipated to enhance patient stratification and advance targeted intervention strategies [70, 74].

## Conclusion

The management of RLDs continues to rely on pharmacological therapies, nutritional optimization, and coordinated multidisciplinary care to stabilize liver function and delay disease progression. Early recognition of these conditions requires heightened awareness and broader access to genetic testing. Advances in molecular biology and genomics are paving the way for precision-based approaches that utilize validated biomarkers for early detection and ongoing monitoring. Long-term correction of inherited defects through gene and RNA therapies is currently under clinical evaluation. Therefore, strengthening international collaborations and ensuring unbiased access to advanced treatments are imperative for improving patient prognosis and enhancing the quality of life for affected individuals.
